# Neural Correlates Underlying General and Food-Related Working Memory in Females with Overweight/Obesity

**DOI:** 10.3390/nu17152552

**Published:** 2025-08-04

**Authors:** Yazhi Pang, Yuanluo Jing, Jia Zhao, Xiaolin Liu, Wen Zhao, Yong Liu, Hong Chen

**Affiliations:** 1 Key Laboratory of Cognition and Personality, Ministry of Education, Southwest University, Chongqing 400715, China; 2 School of Psychology, Southwest University, Chongqing 400715, China; 3 School of Music, Southwest University, Chongqing 400715, China; 4 School of International Studies, Civil Aviation University of China, Tianjin 300300, China; 5 Faculty of Education, Southwest University, Chongqing 400715, China; 6 Research Center of Psychology and Social Development, Faculty of Psychology, Southwest University, Chongqing 400715, China

**Keywords:** overweight/obesity, working memory, high-calorie and low-calorie foods, ERPs

## Abstract

Background/Objectives: Prior research suggest that poor working memory significantly contributes to the growth of overweight and obesity. This study investigated the behavioral and neural aspects of general and food-specific working memory in females with overweight or obesity (OW/OB). Method: A total of 54 female participants, with 26 in the OW/OB group and 28 in the normal-weight (NW) group, completed a general and a food-related two-back task while an EEG was recorded. Results: In the general task, the OW/OB group showed significantly poorer performance (higher IES) than the NW group (*p* = 0.018, *η*^2^ = 0.10), with reduced theta power during non-target trials (*p* = 0.040, *η*^2^ = 0.08). No group differences were found for P2, N2, or P3 amplitudes. In the food-related task, significant group × stimulus interactions were observed. The OW/OB group showed significantly higher P2 amplitudes in high-calorie (HC) versus low-calorie (LC) food conditions (*p* = 0.005, *η*^2^ = 0.15). LPC amplitudes were greater in the OW/OB group for HC targets (*p* = 0.036, *η*^2^ = 0.09). Alpha power was significantly lower in OW/OB compared to NW in HC non-targets (*p* = 0.030, *η*^2^ = 0.09), suggesting a greater cognitive effort. Conclusions: These findings indicate that individuals with OW/OB exhibit deficits in general working memory and heightened neural responses to high-calorie food cues, particularly during non-target inhibition. The results suggest an interaction between reward salience and cognitive control mechanisms in obesity.

## 1. Introduction

The prevalence of overweight (OW) and obesity (OB) is rapidly increasing in China, with a general obesity rate (BMI ≥ 30 kg/m^2^) of 14.0% and abdominal obesity rate (WC ≥ 90 cm for men and ≥80 cm for women) of 31.5% [1]. The obesity issue is further pronounced in children, rising from 5.3% in 1995 to 20.5% in 2014 [2], posing a significant public health threat. Obesity is recognized as a contributory factor to cognitive impairment [3], and various studies have explored its impact on working memory [4,5]. Working memory plays a role in maintaining task-relevant information in the presence of distracting or interfering stimuli [6]. A meta-analysis revealed broad executive function impairments in individuals with OB, including inhibition, decision-making, verbal fluency, cognitive flexibility, and planning. Individuals with OW primarily showed deficits in inhibition and working memory, indicating the vulnerability of working memory to excess weight [7]. Children with obesity exhibit slower performance in working memory tasks [8]. Additionally, studies using food cues found slower reaction times to high-calorie (HC) savory foods during the n-back task, suggesting a potential impairment in working memory performance induced by HC foods [9]. Lower levels of working memory performance are associated with a loss of control over eating, less frequent fruit and vegetable consumption, and increased intake of HC foods [10,11,12,13]. These findings suggest the potential impact of high-calorie foods on working memory, and their potential influence on eating behavior and food choices.

ERP studies indicated that P2 reflects early short-term memory storage and context updating in working memory [14,15]. Research indicated that P2 reflects early attentional processes, such as the detection of salient stimuli and the allocation of attentional resources [14]. Previous studies have shown that individuals with OW/OB exhibit increased P2 amplitudes in food-related tasks [16,17], suggesting enhanced attentional responsiveness to food-related stimuli. N2, related to novelty detection and cognitive control, exhibited lower amplitudes in individuals with OW/OB, indicating lower executive control [16,18]. During a food go/no-go task, N2 amplitudes for HC foods were higher than those for low-calorie (LC) foods [16]. P3, sensitive to working memory demands, exhibited higher amplitudes in individuals with OW/OB during food-related executive function tasks [16,19]. Late positive component (LPC) amplitudes were also higher in the OW/OB group, reflecting the engagement of cognitive resources and consideration of long-term consequences [18,19].

A prior investigation established a link between theta and alpha oscillations and working memory [20]. Theta oscillations have been consistently linked to memory load and executive function. For instance, theta power increases with the number of items held in the working memory [21,22], reflecting the active maintenance of information. In food-related cognitive tasks, low-calorie (LC) foods have been shown to elicit greater theta activity than HC foods, suggesting that HC foods may impair conflict monitoring or cognitive control [23]. Moreover, females with obesity demonstrated decreased theta power compared to those with a normal weight [24], possibly indicating impairments in cognitive regulation [25]. Alpha oscillations, on the other hand, are thought to reflect the inhibition of irrelevant stimuli and the protection of task-relevant information in working memory [26,27]. Notably, alpha power typically decreases as memory load increases, serving as a neural marker of increased cognitive effort [28]. Decreased alpha power has been observed in individuals with OW/OB [29], further supporting the notion of diminished cognitive function in this population.

While previous ERP studies have examined food-related inhibitory control in obesity, few have systematically compared general vs. food-related working memory at both behavioral and neural levels. Moreover, prior literature has suggested potential compensatory neural processes in obesity, such as increased prefrontal activation during cognitive tasks, which might reflect the additional cognitive effort needed to overcome underlying deficits in executive function or cognitive control [30,31]. Including such mechanisms in our investigation provides a more comprehensive and balanced view of cognitive processing in obesity. To bridge this gap, the current study explored the behavioral and neural correlates of general and food-related working memory in individuals with OW/OB and an NW. Building on previous research, P2, N2, P3, and LPC were selected as neural correlates, with the following hypotheses: (1) the OW/OB group would perform inferiorly compared to the NW group during general and food-related n-back tasks; (2) the OW/OB group would exhibit a heightened focus on food-related stimuli, potentially involving higher-order cognitive processes in motivation toward such stimuli, leading to increased P2, P3, and LPC amplitudes during the food-related working memory task. However, impaired conflict monitoring in individuals with OW/OB would be reflected in decreased N2 amplitudes during general and food-related working memory tasks. Additionally, the compromised working memory in the OW/OB group would be evident through a reduction in theta and alpha power. While this study integrates both general and food-specific working memory tasks in the same ERP and time–frequency framework, previous studies have explored domain-specific cognitive processes using ERP methodologies [17,32]. However, this is the first study to combine both types of tasks within a single experimental framework, which offers a more comprehensive understanding of the cognitive mechanisms involved.

## 2. Methods

### 2.1. Participants

Fifty-four participants (all females) were recruited from Southwest University, China. Participants with BMI equal to or greater than 25 kg/m^2^ were included in the OW/OB group (*N* = 26, Mage = 20.71 SDage = 1.15), and those with BMI between 18 and 22 kg/m^2^ were included in the NW group (*N* = 28, Mage = 20.85, SDage = 1.16). A priori power analysis was conducted using G*power (version 3.0). To obtain a power of 0.95 with the alpha level set at 0.05 to observe a medium effect size of 0.25 [30], the minimal sample size of 36 was suggested for mixed-design ANOVA tests. Only female participants were recruited to reduce between-subject variability associated with gender, as prior studies have shown sex-related differences in both neural and behavioral responses to food cues and cognitive control tasks [33,34,35]. This choice was made to improve internal consistency within the sample and reduce potential confounding effects of gender-related heterogeneity. All participants were required to disclose any history of major psychological disorders and reported normal or corrected-to-normal vision. Participants reported not taking any chronic or acute medications. Written informed consent was obtained from all participants prior to their participation. Ethical approval of the study was received from the Southwest University Ethics Committee (IRB No. H22020).

### 2.2. Procedure

Participants were instructed to refrain from consuming any substances, aside from water, for at least 4 h prior to the experiment, in order to standardize intake state [16,18]. After signing informed consent forms, participants proceeded to rate their current levels of hunger and desire to eat. They then continued to complete the general and food-related two-back tasks, with EEG data being recorded simultaneously. The order of the two-back tasks was counterbalanced across participants.

### 2.3. Measures

#### 2.3.1. Hunger and Desire to Eat

Participants rated their hunger and desire to eat on a 100 mm visual analog scale (VAS), rated from “not at all” to “very high”.

#### 2.3.2. Working Memory Tasks (Two-Back Tasks) (Figure 1)

In this study, the general two-back and food-related two-back tasks were employed to separately investigate general working memory and food-related working memory.

**Figure 1:**
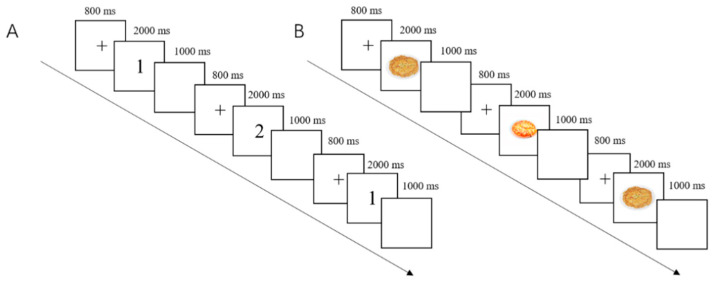
The general (**A**) and food-related (**B**) two-back tasks used in the present study.

#### 2.3.3. General Two-Back Task (Figure 1A)

In the general two-back task (Figure 1A), the stimuli consisted of numbers 1 to 9. Participants’ objective was to determine whether the current number shown on the screen matched the number presented two trials before. Responses involved pressing “F” with their left hand for identical numbers and “J” for different numbers with their right hand (Figure 1) [36], and the mapping between response keys and stimulus types was fixed throughout the task. The task encompassed 60 trials, including 30 target stimuli. Each trial starts with an 800 ms fixation on the screen, followed by the presentation of the stimuli, which would be displayed on the screen for 2000 ms or until the participants made a response. Finally, a blank screen would be displayed for 1000 ms.

#### 2.3.4. Food-Related Two-Back Task (Figure 1B)

Food-related two-back task (Figure 1B) was modified from the regular two-back task using stimuli consisting of food pictures from a food picture library previously developed by our research team [37,38]. Images were presented on a white background, and matched across HC and LC categories for size, color saturation, brightness, contrast, and visual complexity to minimize perceptual confounds. The task comprised 30 HC and 30 LC food pictures. Participants were asked to determine whether the current food picture matched the one two trials prior. Responses were pressing “F” for the same picture with the left hand and “J” for different pictures with the right hand, and the mapping between response keys and stimulus types was fixed throughout the task. The task consisted of 120 trials, including 60 target stimuli (30 HC and 30 LC food pictures). Similar to the general task, each trial initiated with an 800 ms fixation on the screen, and stimuli were displayed for 2000 ms or until participants responded. The trial would also end with a blank screen appearing for 1000 ms on the monitor. The stimulus sequences for both the general and the food-related tasks were randomized within each task to minimize any sequence effects.

The general and food-related tasks were developed and administered using the E-Prime 3.0 software, with stimuli presented on a 24-inch display. Participants were required to respond as promptly and accurately as possible. Prior to the official task, they completed 20 practice trials with feedback, requiring a minimum accuracy rate of 60%. All participants were successful at meeting the 60% threshold. Note that the general and food-related tasks utilized different stimuli (digits vs. food images), which was intentional to dissociate domain-general and domain-specific working memory processes.

### 2.4. Behavioral Analysis

In the current study, behavioral performance was measured using the inverse efficiency score (IES), which balances accuracy and speed (i.e., IES = RT/ACC). A higher IES indicates lower efficiency [39,40]. Trials with incorrect responses or reaction times below 100 ms were excluded for the final analysis. Participants whose scores exceeded ±3 SDs were excluded, resulting in a final sample of 51 participants (25 overweight and 26 normal weight). For the food-related two-back task, all participants (26 overweight and 28 normal weight) were included in the final analysis.

For the general two-back task, we conducted repeated-measures ANOVAs [2 (group: OW/OB and NW) × 2 (stimuli: targets and non-targets)], utilizing group as a between-subjects factor and stimuli as a within-subjects factor. Furthermore, we performed repeated-measures ANOVAs for the food-related two-back task [2 (group: OW/OB and NW) × 2 (food: HC and LC) × 2 (stimuli: targets and non-targets)], incorporating group as a between-subjects factor and food and stimuli as within-subjects factors.

All statistical analysis were executed by SPSS 27.0 (IBM, New York, NY, USA), with adjustments of *p*-values for sphericity utilizing the Greenhouse–Geisser method. The post-hoc *t*-tests with Bonferroni correction were employed for multiple comparisons.

### 2.5. EEG Recording and Analysis

Brain electrical activities were recorded using an elastic cap (Neuroscan, Charlotte, NC, USA) with 32 scalp sites equipped with tin electrodes. EEG data were recorded using a reference electrode (REF) placed at FPz and a ground electrode (GRD) positioned at FCz. Electrooculogram (EOG) signals, representing eye movements, were recorded with an electrode on the intraorbital area of the left eye. Inter-electrode impedance was maintained below 5 kΩ during gel application and recording.

EEG data processing utilized MATLAB R2022a with the EEGLAB toolbox [36]. Data underwent bandpass finite impulse response (FIR) filtering between 0.1 and 30 Hz, re-referencing the left and right mastoids. Trials (−200 ms to 1000 ms) were segmented according to stimulus marks, baseline-corrected (−200 ms to 0 ms), and inspected for data quality. Abnormal fluctuation trials were eliminated, and bad channels were corrected by averaging adjacent electrodes’ amplitudes.

In order to remove interference factors, such as eye movements and electrocardiography, independent components analysis (ICA) was performed using the built-in runica function in EEGLAB. This procedure allowed us to remove eye movement and muscle artifacts. Specifically, we identified independent components corresponding to artifacts and removed them based on visual inspection, ensuring minimal data loss. Artifact rejection thresholds were set at ±100 µV for all channels. Fz was selected due to its established sensitivity to cognitive control and conflict processing in working memory tasks, and to minimize contamination from motor responses [41]. Time windows for ERP activities based on grand-averaged data were as follows: General two-back task: P2, 150–200 ms; N2, 200–250 ms; P3, 300–400 ms; and LPC, 500–1000 ms. Food-related two-back task: P2, 150–190 ms; N2, 190–250 ms; P3, 250–400 ms; and LPC, 500–1000 ms. The time windows selected for ERP components were based on prior studies involving food-related or cognitive-control paradigms [42], as well as visual inspection of the grand-average waveforms in the current data.

Time-frequency analysis utilized a windowed Fourier transform (WFT) with a 250 ms width Hanning window. Theta (4–8 Hz, 200–400 ms) and alpha (8–13 Hz, 300–600 ms) brain rhythmic activities were selected for the general two-back task, while theta (4–8 Hz, 100–300 ms) and alpha (8–13 Hz, 400–700 ms) were chosen for the food-related two-back task. At the individual subject level, a baseline correction was applied using the pre-stimulus interval (from −200 ms to 0 ms prior to stimulus onset) to calculate the change in power according to the following formula:

TFD (t, f) = P(t, f) − R(f)

where P(t, f) = ∣F(t, f)∣2P(t, f) = |F(t, f)|^2P(t, f) = ∣F(t, f)∣2 is the power spectral density at a specific time–frequency point, and R(f)R(f)R(f) is the averaged power spectral density within the pre-stimulus reference interval (−200 to 0 ms) for each frequency.

Repeated-measures ANOVAs were conducted for both tasks on the amplitudes of P2, N2, P3, LPC, theta, and alpha power. ERP components were selected based on prior work linking them to cognitive processes relevant to food-related and executive control paradigms. P2 is associated with early attentional orienting, particularly toward salient or emotionally relevant stimuli [43]. N2 is commonly interpreted as reflecting conflict monitoring and response inhibition [42]. P3 has been linked to attentional resource allocation and working memory updating [44], while LPC reflects sustained processing and motivational evaluation [45,46]. Theta power increases are often interpreted as markers of cognitive effort or top-down control, while decreases in alpha power have been related to increased mental workload and attentional engagement [47,48]. For the general two-back task: [2 (group: OW/OB and NW) × 2 (stimuli: targets and non-targets)]. For the food-related two-back task: [2 (group: OW/OB and NW) × 2 (food: high-calorie and low-calorie) × 2 (stimuli: targets and non-targets)]. The statistical analyses were performed using SPSS 27.0, with adjustments of *p*-values for sphericity using the Greenhouse–Geisser method. Post-hoc *t*-tests with Bonferroni correction were applied for multiple comparisons.

## 3. Results

### 3.1. Hunger and Desire to Eat

Independent-sample *t*-tests revealed that no significant difference was observed between the OW/OB and the NW group in hunger, *t* (52) = 0.19, *p* = 0.85, nor in desire to eat, *t* (52) = 0.72, *p* = 0.48.

### 3.2. Behavioral Results

#### 3.2.1. General Two-Back Task

**Figure 2:**
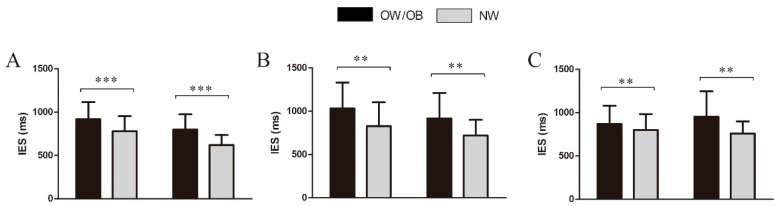
Behavioral results in the general and food-related two-back tasks. (**A**) The general working memory performance; (**B**) The high-calorie food-related working memory performance; (**C**) The low-calorie food-related working memory performance. Note: IES, inverse efficiency score; OW/OB, individuals with overweight/obesity; NW, normal-weight individuals; *** *p* < 0.001; ** *p* < 0.01.

Results on the IES (Figure 2) revealed a main effect of group, *F* (1, 49) = 15.47, *p* < 0.001, partial *η*^2^ = 0.24, and the post hoc *t*-test revealed that the IES in the OW/OB group was significantly greater than that in the NW group. Results on the IES also revealed a main effect of stimuli, *F* (1, 49) = 31.39, *p* < 0.001, partial *η*^2^ = 0.39, and the post hoc *t*-test revealed that the IES in the non-targets was greater those that in the targets. All comparisons shown have been corrected for multiple comparisons using the Bonferroni method.

#### 3.2.2. Food-Related Two-Back Task

Results on the IES (Figure 2) revealed a main effect of group, *F* (1, 52) = 10.02, *p* = 0.003, partial *η*^2^ = 0.16, and the post hoc *t*-test showed that the IES in the OW/OB group was significantly greater than that in the NW group. Results on the IES indicated an interaction of food and stimuli, *F* (1, 52) = 7.32, *p* = 0.009, partial *η*^2^ = 0.12. The simple effect analysis revealed no significant difference in the IES between the HC foods and the LC foods in the non-targets, *p* = 0.06. All comparisons shown have been corrected for multiple comparisons using the Bonferroni method.

### 3.3. Neural Correlate Results

#### 3.3.1. General Two-Back Task (Figure 3)

Results on P3 amplitudes revealed a main effect of stimuli, *F* (1, 49) = 20.6, *p* < 0.001, partial *η*^2^ = 0.30, and the post hoc *t*-test revealed that P3 amplitudes in the targets were greater than those in the non-targets. We did not find any difference between the OW/OB and the NW group in P2, N2, LPC, and theta power, with all *p* > 0.05.

Results on alpha (Figure 4) revealed an interaction of group and stimuli, *F* (1, 49) = 6.10, *p* = 0.02, partial *η*^2^ = 0.11. The simple effect analysis showed that the alpha power in the targets was significantly greater than that in the non-targets in the NW group, *F* (1, 49) = 8.62, *p* = 0.005, partial *η*^2^ = 0.15. No significant difference was observed between the targets and non-targets in the OW/OB group, *F* (1, 49) = 0.34, *p* = 0.56, partial *η*^2^ = 0.007. No significant difference was observed between the OW/OB and the NW group, *p* = 0.86.

**Figure 3:**
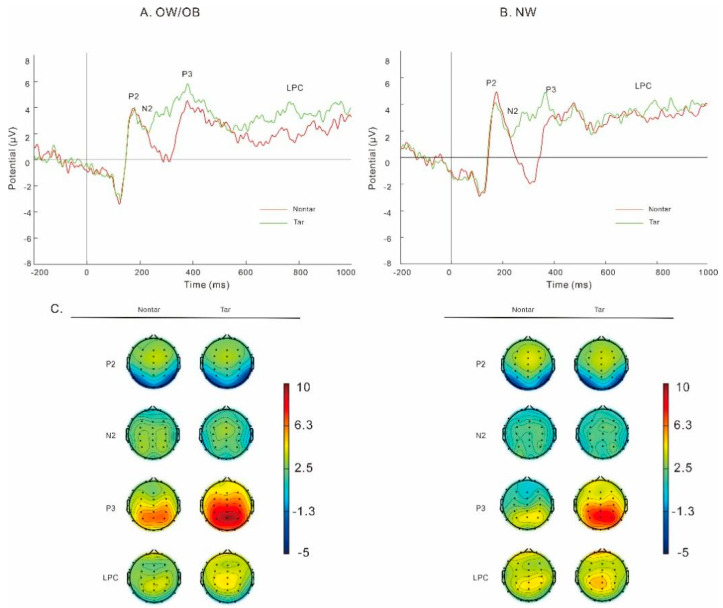
The amplitudes (**A**,**B**) and topography plots (**C**) for individuals with overweight/obesity (**A**) and normal weight (**B**) in the general two-back task. Note: OW/OB, overweight/obesity; NW, normal weight; Nontar, nontarget; Tar, target.

**Figure 4:**
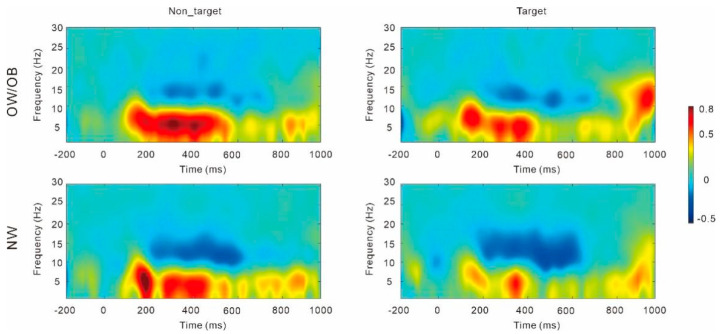
Time–frequency analysis for the individuals with overweight/obesity and normal weight in the general two-back task. Note: OW/OB, overweight/obesity; NW, normal weight.

#### 3.3.2. Food-Related Two-Back Task (Figure 5)

##### P2

Results on P2 amplitudes revealed a main effect of food, *F* (1, 52) = 8.95, *p* = 0.004, partial *η*^2^ = 0.15, and the post hoc *t*-test revealed that P2 amplitudes in the HC foods were greater than those in the LC foods.

**Figure 5:**
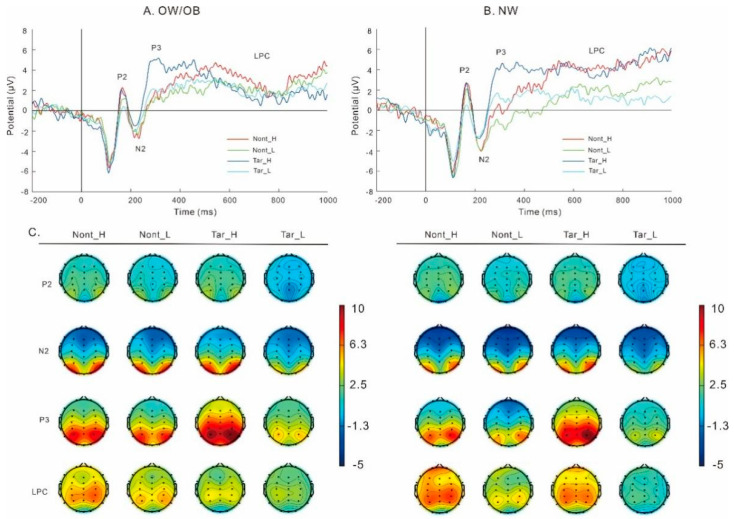
The amplitudes (**A**,**B**) and topography plots (**C**) for individuals with overweight/obesity (**A**) and normal weight (**B**) in the food-related two-back task. Note: OW/OB, overweight/obesity; NW, normal weight; Nont_H, nontarget_high-calorie foods; Nont_L, nontarget_low-calorie foods; Tar_H, target_high-calorie foods; Tar_L, target_low-calorie foods.

##### N2

Results on N2 amplitudes showed a main effect of stimuli, *F* (1, 52) = 4.93, *p* = 0.03, partial *η*^2^ = 0.09, and the post hoc *t*-test revealed that N2 amplitudes in the non-targets were greater than those in the targets. Results on N2 amplitudes also revealed a main effect of group, *F* (1, 52) = 3.94, *p* = 0.05, partial *η*^2^ = 0.07, and the post hoc *t*-test revealed that N2 amplitudes in the OW/OB group were lower than those in the NW group.

##### P3

Results on P3 amplitudes revealed a main effect of food, *F* (1, 52) = 20.33, *p* < 0.001, partial *η*^2^ = 0.28, and the post hoc *t*-test revealed that P3 amplitudes in the HC foods were higher than those in the LC foods. Results on P3 amplitudes revealed a main effect of stimuli, *F* (1, 52) = 22.73, *p* < 0.001, partial *η*^2^ = 0.30, and the post hoc *t*-test revealed that P3 amplitudes in the targets were higher than those in the non-targets. No significant interaction between group and stimuli was found, *F* (1, 52) = 3.45, *p* = 0.069, partial *η*^2^ = 0.06.

##### LPC

Results on LPC amplitudes revealed a main effect of food, *F* (1, 52) = 22.62, *p* < 0.001, partial *η*^2^ = 0.30, and the post hoc *t*-test showed that LPC amplitudes in the HC foods were higher than those in the LC foods.

##### Theta

**Figure 6:**
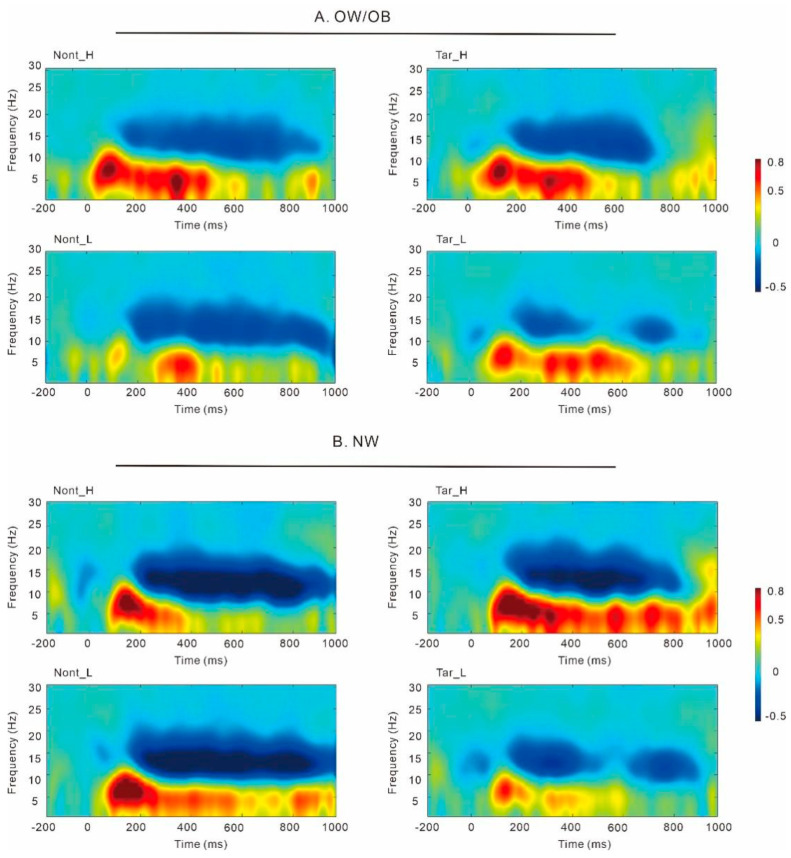
Time–frequency analysis for the individuals with overweight/obesity (**A**) and normal weight (**B**) in the food-related two-back task. Note: OW/OB, overweight/obesity; NW, normal weight; Nont_H, nontarget_high-calorie foods; Nont_L, nontarget_low-calorie foods; Tar_H, target_high-calorie foods; Tar_L, target_low-calorie foods.

Results on theta power (Figure 6) revealed a main effect of group, *F* (1, 52) = 5.25, *p* = 0.026, partial *η*^2^ = 0.09, and the theta power in the OW/OB group was significantly lower than that in the NW group. The results also revealed a main effect of food, *F* (1, 52) = 14.66, *p* < 0.001, partial *η*^2^ = 0.22, and the theta power in the HC foods was higher than that in the LC foods.

##### Alpha

Results on alpha power (Figure 6) revealed a main effect of stimuli, *F* (1, 52) = 6.16, *p* = 0.02, partial *η*^2^ = 0.11, and the alpha power in the targets was significantly higher than that in the non-targets. The results also revealed a main effect of food, *F* (1, 52) = 11.81, *p* = 0.001, partial *η*^2^ = 0.19, and the alpha power in the low-calorie foods was higher than that in the HC foods. Importantly, the results revealed a main effect of group, *F* (1, 52) = 5.79, *p* = 0.02, partial *η*^2^ = 0.10, and the alpha power in the OW/OB group was significantly lower than that in the NW group.

To provide a clearer overview of group- and condition-wise neural responses, descriptive statistics (mean ± standard deviation) and effect sizes (partial eta squared or Cohen’s *d*) for ERP components (P2, P3, and LPC) are presented in Table 1 and Table 2. These data reflect the amplitude differences across groups and stimulus types and complement the inferential statistics reported above.

##### Correlation Between Behavioral and EEG Measures

To further examine the relationship between behavioral performance and EEG indices during the food-related working memory task, Pearson correlations were conducted between the IES and relevant ERP components (P2, N2, P3, LPC) as well as theta and alpha power.

**Figure 7:**
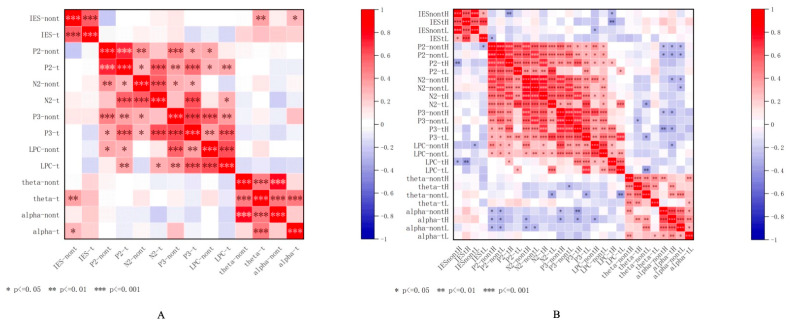
Correlation between behavioral and EEG measures in the general two-back task (**A**) and food-related two-back task (**B**). Note: IES, inverse efficiency scores; nont, nontarget; t, target. *** *p* < 0.001; ** *p* < 0.01; * *p* < 0.05.

The results (Figure 7) indicated several significant correlations: In the general two-back task (Figure 7A), significant positive correlations were found between IES-t and IES-nont, r(51) = 0.583, *p* < 0.05; P2-t and N2-nont, r(51) = 0.412, *p* < 0.05; N2-t and P2-t, r(51) = 0.574, *p* < 0.05; N2-t and LPC-t, r(51) = 0.340, *p* < 0.05; P3-nont and N2-t, r(51) = 0.637, *p* < 0.05; P3-nont and P3-t, r(51) = 0.645, *p* < 0.05; P3-nont and LPC-t, r(51) = 0.361, *p* < 0.05; P3-t and LPC-t, r(51) = 0.577, *p* < 0.05; Theta-t and LPC-t, r(51) = 0.626, *p* < 0.05; Theta-t and Theta-nont, r(51) = 0.362, *p* < 0.05; Alpha-nont and Alpha-t, r(51) = 0.281, *p* < 0.05; Theta-nont and Theta-t, r(51) = 0.495, *p* < 0.05; Alpha-t and Theta-t, r(51) = 0.509, *p* < 0.05; and Alpha-t and Theta-nont, r(51) = 0.509, *p* < 0.05.

In the food-related two-back task, significant positive correlations were found between IES-t and IES-nont, r(54) = 0.593, *p* < 0.05; IES-t and P2nontL, r(54) = 0.348, *p* < 0.05; IES-t and N2nontL, r(54) = 0.351, *p* < 0.05; IES-t and P3nontL, r(54) = 0.393, *p* < 0.05; IES-t and P3tL, r(54) = 0.342, *p* < 0.05; IES-t and LPCnontL, r(54) = 0.351, *p* < 0.05; IES-t and LPCtL, r(54) = 0.324, *p* < 0.05; P2tL and P3nontL, r(54) = 0.417, *p* < 0.05; P3nontL and P2tL, r(54) = 0.417, *p* < 0.05; P3nontL and N2nontL, r(54) = 0.374, *p* < 0.05; P3tL and P2tL, r(54) = 0.416, *p* < 0.05; LPCtL and P3tL, r(54) = 0.341, *p* < 0.05; Theta-t and LPCtL, r(54) = 0.360, *p* < 0.05; Alpha-t and LPCtL, r(54) = 0.322, *p* < 0.05; Alpha-t and LPCnontL, r(54) = 0.365, *p* < 0.05; Alpha-t and Theta-t, r(54) = 0.363, *p* < 0.05; and Alpha-t and Theta-nont, r(54) = 0.323, *p* < 0.05. Significant negative correlations were found between LPCnontL and P3tL, r(54) = −0.336, *p* < 0.05; LPCnontL and LPCtL, r(54) = −0.321, *p* < 0.05; and LPCtL and IES-t, r(54) = −0.324, *p* < 0.05. None of the other results are significant.

## 4. Discussion

In this study, our hypotheses were partially confirmed. As speculated, individuals with OW/OB demonstrated less optimal performance in both general and food-related two-back tasks, evident in the significantly higher IES within the OW/OB group compared to the NW group. Nevertheless, no differences in the neural correlates were observed between the two groups during the general two-back task. However, results from the food-related two-back task supported the present hypotheses. Specifically, the N2 amplitudes, theta, and alpha power in the OW/OB group were lower than those in the NW group. Furthermore, the P2, P3, and LPC amplitudes were higher for HC foods compared to LC foods. Theta power exhibited greater levels in HC foods than in LC foods, while alpha power showed the opposite pattern. Although both tasks revealed reduced behavioral performance in individuals with OW/OB, only the food-related task elicited neural differences. This suggests that food-specific stimuli may more effectively engage neural systems involved in attention and cognitive control in this population, reflecting a higher motivational salience.

The results showed that the amplitudes of P2, P3, and LPC were higher for HC foods than for LC foods, and this might due to the fact that HC foods elicited increased attentional engagement and deeper cognitive processing. P2 is linked to attention processing during stimulus evaluation [47], while P3 and LPC are associated with the allocation of cognitive resources [48,49], with LPC being related to higher-order cognitive processing involving motivation or emotion [45,46]. Thus, the elevated responses to HC foods may reflect an association with increased salience or emotional engagement, potentially prompting stronger top-down cognitive responses. This is consistent with prior findings that HC food stimuli are associated with poorer executive performance, which may reflect greater attentional resource demands [23]. However, there was no significant difference between HC and LC foods in terms of behavioral performance. Similarly, the difference between non-targets and targets in the HC condition was also non-significant, suggesting possible effects that require confirmation in future studies.

Regarding group disparities at the behavioral level, we noted that the IES in OW/OB group was significantly greater than that in the NW group during both general and food-related working memory tasks, indicating poorer performance by the OW/OB group in these tasks. Previous studies [4,8,50] focusing on general working memory in the OW/OB group consistently reported poorer performance compared to the NW group. Moreover, working memory and inhibitory control interact during cognitive tasks, with inhibitory control supporting working memory by filtering irrelevant information [6,51,52]. Deficiencies in working memory in individuals with OW/OB may be influenced by inhibitory control, as demonstrated in our previous studies indicating poorer inhibitory control [18,19]. Future experimental tasks could delve into the causal relationship between inhibitory control and working memory in cognitive tasks performed by individuals with OW/OB.

Anterior N2 is linked to cognitive control, potentially indicating the detection and monitoring of response conflict [53,54]. The diminished N2 amplitudes observed in the OW/OB group may reflect an association with reduced efficiency in cognitive control processes, which potentially linked to increased attentional capture by salient food cues. The OW/OB group may necessitate more time for the working memory task, consistent with the observed group difference in behavioral performance. P3 amplitudes reflect processing intensity [55], and the increased P3 amplitudes in individuals with OW/OB may suggest a heightened allocation of cognitive resources and elevated reward sensitivity to food stimuli. Differences in P2, P3, and LPC amplitudes between HC and LC foods may reflect an attentional or cognitive bias associated with HC food stimuli. Reduced N2 and heightened P3 amplitudes for targets compared to non-targets, aligning with prior studies [17,56], hint at diminished target monitoring and enhanced cognitive resource allocation for target stimuli. Theta and alpha power in the OW/OB group, which were lower than in the NW group, support previous findings [24,29]. Despite variations in some neural correlates (P3 and alpha power) in the general working memory task, no group differences surfaced in neural correlates during this task. This leads us to cautiously propose that differences in working memory neural correlates between individuals with OW/OB and with NW in this study may be food-specific. Food-related cognitive tasks might provide a more suitable context for exploring cognitive function and potential neural correlates in individuals with obesity.

The correlation findings provide further support for the role of specific neural components in food-related working memory. The results revealed that several ERP components (e.g., P2, N2, P3, and LPC) and oscillatory activities (theta and alpha power) were significantly associated with behavioral efficiency. In particular, higher LPC amplitudes and greater theta/alpha power were linked to better task performance, suggesting that stronger late-stage cognitive processing and neural engagement may support more efficient responses. These findings provide converging evidence that both ERP and time–frequency markers reflect meaningful individual differences in executive functioning during food-related cognitive tasks.

Additionally, we acknowledge that the relationship between obesity and cognitive function is likely bidirectional. While impaired executive functions may contribute to overeating and weight gain, emerging longitudinal evidence suggests that obesity-related neurobiological changes—such as reduced prefrontal volume or altered dopaminergic pathways—can in turn impair cognitive control over time [31,57]. This dynamic interplay implies a reciprocal feedback loop, wherein obesity and cognitive deficits reinforce each other across development. Longitudinal studies support this more complex directional model [58,59].

We acknowledge the possibility of reverse causality in the relationship between obesity and cognitive function. That is, rather than cognitive impairments contributing to obesity, it is also plausible that obesity-related neurobiological changes—such as reduced prefrontal volume or altered dopamine signaling—may lead to poorer executive function over time [31,57]. Longitudinal studies are needed to disentangle the directionality of this relationship.

Several limitations in this study warrant acknowledgment. Firstly, the stimuli used in the general working memory task involve numbers, while the food working memory task utilizes food pictures, hindering a direct comparison of behavioral and neural differences between the two tasks. Future studies could investigate general working memory using neutral stimuli (e.g., flowers) and compare them to food-related working memory. Secondly, the exclusive focus on females in this study limits the generalizability of the conclusions. Future research should consider gender differences to enhance the broader applicability of findings. Third, the use of an all-female sample, while methodologically intended to reduce variability and increase internal validity, limits the generalizability of our findings. Given known sex differences in reward sensitivity, hormonal influences, and prefrontal–limbic responses to food stimuli [60,61], future studies should include mixed-gender samples or explicitly test for gender as a moderating variable. Additionally, despite meeting an a priori power analysis threshold, the current sample size may still limit the detection of subtle neural effects typically investigated with ERP methods. Future studies should consider larger samples to confirm the observed results. Another limitation of the current study is the absence of objective screening for metabolic indicators such as blood glucose and lipid levels, which are known to influence cognitive function. Although all participants were young university students who reported no known health issues, the potential influence of undiagnosed metabolic conditions cannot be fully excluded. Future research should consider including medical assessments to better characterize participants’ health status. In addition, although hunger states were controlled, other influential factors such as restrained or emotional eating tendencies, as well as socioeconomic status, were not measured. Future studies should account for these variables to clarify their potential influence on task performance. Moreover, although BMI is a commonly used measure of obesity, it has limitations in distinguishing fat distribution and body composition [62]. Future studies should consider incorporating additional metrics such as waist-to-hip ratio to better capture obesity-related differences in cognitive functioning. Lastly, as a cross-sectional study, the relationship between working memory and eating behaviors in individuals with OW/OB remains unconfirmed. Future longitudinal research designs could elucidate the causal relationship between working memory and eating behavior in this population.

## 5. Conclusions

In conclusion, this study demonstrates that individuals with OW/OB are associated with poorer performance on both general and food-related working memory tasks compared to the NW group. Individuals with OW/OB also exhibited neural responses indicative of heightened attentional and reward processing towards HC foods, potentially allocating more cognitive resources when processing food-related stimuli. In contrast to the NW group, the OW/OB group appeared to experience greater difficulty recruiting cognitive resources to detect differentiate between targets and non-targets specifically within the food-related working memory context. While group differences in ERP components and time–frequency measures were most pronounced in the food-related task, we caution against interpreting these effects as evidence of domain-specific working memory impairment. Rather, these neural differences may reflect heightened sensitivity to the salience, motivational value, or emotional valence of high-calorie food cues. The increased P2, P3, and LPC amplitudes, along with the altered theta and alpha power, could index attentional bias or affective processing differences rather than purely executive dysfunction. Longitudinal and multimodal studies are needed to determine whether these patterns are consistent across time, predictive of behavior, and causally linked to weight regulation.

## Figures and Tables

**Table 1:** Descriptive statistics (mean ± SD) and effect sizes for behavioral (IES) and ERP measures across groups and conditions in the general two-back task.

	OW/OB (M ± SD; *N* = 25)		NW (M ± SD; *N* = 26)	
	Nontar	Tar	Nontar	Tar
IES	917.49 (198.61)	797.69 (176.39)	779.95 (173.40)	620.39 (115.19)
P2	3.08 (2.95)	2.98 (4.05)	3.73 (3.09)	3.41 (3.59)
N2	2.03 (2.98)	2.50 (3.28)	1.68 (3.61)	1.94 (4.43)
P3	2.43 (4.73)	4.71 (4.74)	0.69 (3.96)	3.69 (5.88)
LPC	1.97 (5.64)	3.14 (4.57)	2.94 (3.67)	3.29 (4.47)
theta	0.66 (0.74)	0.48 (0.55)	0.44 (0.62)	0.32 (0.52)
alpha	0.06 (0.38)	−0.06 (0.66)	−0.10 (0.59)	−0.15 (0.59)

Note: OW/OB, overweight/obesity; NW, normal weight; M, mean; SD, standard deviation; nont, nontarget; tar, target; IES, inverse efficiency scores.

**Table 2:** Descriptive statistics (mean ± SD) and effect sizes for behavioral (IES) and ERP measures across groups and conditions in the food-related two-back task.

	OW/OB (M ± SD; *N* = 26)				NW (M ± SD; *N* = 28)			
	NontarH	TarH	NontarL	TarL	NontarH	TarH	NontarL	TarL
IES	1032.32 (554.19)	915.52 (293.65)	869.51 (210.27)	951.41 (294.52)	828.29 (274.64)	719.17 (181.00)	799.63 (183.15)	759.23 (140.20)
P2	1.79 (5.01)	2.07 (5.78)	0.84 (4.19)	−0.56 (3.46)	0.29 (4.94)	0.31 (5.92)	−0.04 (4.38)	−0.48 (3.34)
N2	−1.07 (5.33)	0.07 (4.87)	−1.16 (4.31)	−1.64 (3.88)	−3.88 (5.28)	−3.09 (5.93)	−3.92 (5.32)	−2.29 (3.82)
P3	2.44 (5.55)	4.67 (5.05)	0.98 (4.05)	1.24 (4.34)	−0.19 (5.77)	2.87 (6.69)	−1.59 (5.35)	1.01 (3.79)
LPC	3.06 (5.45)	2.09 (4.69)	1.43 (3.35)	0.78 (4.29)	4.15 (4.99)	4.45 (5.38)	1.73 (4.18)	1.50 (3.98)
theta	0.45 (0.61)	0.37 (0.76)	0.26 (0.55)	0.38 (0.57)	0.75 (0.86)	1.02 (1.13)	0.67 (1.10)	0.45 (0.56)
alpha	0.02 (0.37)	−0.01 (0.35)	−0.09 (0.46)	0.03 (0.24)	−0.61 (1.04)	−0.44 (0.94)	−0.44 (0.88)	−0.08 (0.72)

Note: OW/OB, overweight/obesity; NW, normal weight; M, mean; SD, standard deviation; nont, nontarget; tar, target; IES, inverse efficiency scores.

## Data Availability

The data presented in this study are available on request from the corresponding author due to privacy reasons.
